# A Unified Analytical Method Greenness Score (*uAMGS*) Quantifies How Microscopic Imaging Is Greener Than Conventional Liquid Chromatography

**DOI:** 10.1021/acssuschemeng.6c02093

**Published:** 2026-06-06

**Authors:** Kotomi Inaba, Ricardo Monge Neria, Rachel A. Saylor, Lydia Kisley

**Affiliations:** † Department of Chemistry and Biochemistry, 6167 Oberlin College, Oberlin, Ohio 44074, United States; ‡Department of Physics and §Department of Chemistry, 2546 Case Western Reserve University, Cleveland, Ohio 44106, United States

**Keywords:** greenness metrics, chromatography, chemical separations, single-molecule microscopy, high-performance liquid chromatography, minaturization

## Abstract

Green chemistry is a set of principles for assessing, developing, and implementing methods that are safer, more efficient, and less detrimental to the environment. The analytical method greenness score (*AMGS*) is one of many metrics that attempt to evaluate traditional liquid chromatography (LC) based on the energy consumption of the instrument and the safety, health risks, and environmental impact of the solvents employed. Unfortunately, in practice, the *AMGS* is primarily focused on traditional separation methods in the pharmaceutical industry and is not amenable to cutting-edge separation science, including miniaturization. To broaden this scope, the unified Analytical Method Greenness Score (*uAMGS*) is presented here, which clarifies and expands on the underlying mathematics and incorporates both dimensional and uncertainty analysis, enabling its application to a broader range of analytical techniques. The *uAMGS* is used to compare the greenness of two distinct methods: single-molecule microscopy (SMM) and high-performance liquid chromatography (HPLC), which were used to collect equivalent data. *uAMGS* determines that SMM is significantly greener than HPLC due primarily to decreased solvent consumption. Overall, the *uAMGS* should allow chemists ranging from undergraduates to industrial PhDs to assess the greenness of a wide range of separations.

## Graphical abstract

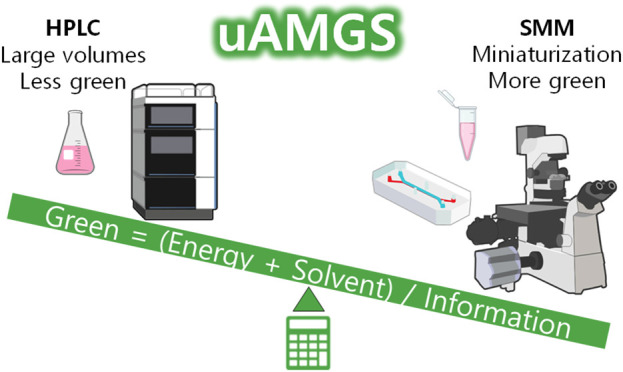

## Introduction

Experimentally evaluating and screening chromatographic separations is an energy-intensive process. For example, in the pharmaceutical industry, the use of chromatography to bring a drug to market requires a large amount of energy, solvent, and time. In the drug discovery phase, libraries of ∼10^3^ to 10^5^ molecules may be synthesized.^1^ Each compound may need to be separated to low (∼85%) purity or analyzed by liquid chromatography (LC)–mass spectrometry (MS) before further activity testing.^2^ LC conditions in these separations are rather fixed (e.g., acid, neutral, and base gradient mobile phases with a single stationary phase) and can have short separation times (<2 min), yet the energy use can be high, given the size of combinatorial libraries and including the cycling time required for column equilibration, washing, and sample preparation for injection.^2^ Next, the pharmaceutical science phase develops analytical methods for separating promising drug candidates iteratively over a large range of LC parameters (e.g., 10s of different stationary phases which sometimes involves the development of custom phases, solvents, flow rates, etc., for 10^2^ molecules).^3^ This extensive LC method development can take weeks or months for a single compound. Finally, the preclinical and clinical phases optimize the separation of a single identified compound to high-quality control standards on different LC instruments.^4,5^ Separation of chiral compounds^6^ or biologics^7^ further complicates the LC method development due to the complex nature of the analytes. Even with automation and high-throughput technologies,^8,9^ liters of solvents per day and energy from 24/7 instrument operation may be used by LC over the timeline of developing a single drug.^10^ Therefore, traditional bulk chromatographic separations lead not only to low greenness,^11,12^ but also to enormous cost in the industries in which it is applied, spanning academia, (bio)­pharma, energy and chemicals, environmental science, and food and beverage production.

The Analytical Method Greenness Score (*AMGS*) has been introduced as a metric to quantitatively compare the greenness of chromatography methods.^13^ Building off of earlier work considering solvent use in high-performance liquid chromatography (HPLC),^14,15^ Hicks and other representatives of the ACS Green Chemistry Institute (GCI) Pharmaceutical Roundtable, in 2019 introduced the *AMGS* metric. The *AMGS* metric considers the contributions of the chromatography instrument energy use and solvent safety and energy demand as a quantitative benchmark to compare separation methods in a noncompetitive manner.^13^ Functionally, the calculator has a drop-down menu with techniques including HPLC, LC-MS, NPLC, Prep LC-MS, Prep SFC, SFC, SFC-MS, UPC2, and UPLC. In practice, the *AMGS* method is therefore primarily specific to traditional liquid or sub/super-critical fluid separations, in contrast to other analytical greenness scores that are more broadly applied to all instrumentation, yet often require a subjective assessment.^16^ −^17,18,19,20,21^ For example, the Analytical GREEnness (AGREE) metric uses specific principles of green chemistry, each normalized on a 0–1 scale, which are then combined for the final AGREE value.^17^ While this process provides an overall qualitative indicator of a method’s greenness, it is inherently not as quantitative as other greenness calculations. In contrast, the *AMGS* is rooted in physical measurements (e.g., solvent volumes, instrument energy consumption) and succeeds in making relative comparisons between objective experimental values of different run times, solvents, and columns that may be used to separate the same set of analytes. Further, the involvement of eight different multinational pharmaceutical companies in the original report and an accessible online calculator^22^ shows that the metric can be adopted by industry as a quick assessment tool for relative comparisons.

Unfortunately, the application of the *AMGS* beyond industry has been limited by the mathematics and the assumption that the metric will only be used for standard commercial liquid chromatography (LC)- or super/subcritical fluid chromatography (SFC)-based instrumentation.^16^ Citation indexing services indicate 100–200 manuscripts have referred to *AMGS*. Specifically, of the 116 papers citing Hicks et al., as accessed through Scifinder in July 2025, ∼30% used some version of the *AMGS* to quantify the greenness of their method, either using the *AMGS* calculator or, less frequently, a modification of the original equations.

As separations advance in part via miniaturization approaches, including microfluidic-based and custom-built instrumentation,^25–30^ the *AMGS* becomes less applicable to the variety of methods in the field. Many of these advances are geared toward faster analysis of smaller volume samples, and as a result, less energy is consumed due to both lower instrument-on time and lower total volume solvent requirements. These types of techniques are not available in the online *AMGS* calculator, leaving fundamental challenges to employ *AMGS* and in comparing *AMGS* values across different techniques and reports.

Additionally, there are specific limitations in the *AMGS*, the first of which are the errors found in the underlying mathematics of the equation, including a multiplicative factor instead of a geometric mean and a misplaced bracket.^23,24^ To the best of our knowledge, these errors have not been corrected in the original report, and it is unclear how the online calculator applies these formulas. These errors and lack of clarity make it difficult to apply *AMGS* without using the online calculator. Second, the resulting *AMGS* values are reported as a single number without any measure of the precision of the score, and the number of significant figures appears to be arbitrarily selected. Based on the authors’ note of a scaling factor that “was about 40%” for the instrument energy use,^13^ it is clear that there could be significant unreported uncertainties in the *AMGS*. Quantitative chemistry requires precision of a metric to understand the statistical significance in the comparison of values across different methods or reports. Overall, clarifying the underlying mathematics of *AMGS*, incorporating uncertainty analysis, and further expanding its use as a metric to understand the improvement in greenness through miniaturization or the development of new methods and instrumentation would be a welcome addition to the field.

Single-molecule microscopy (SMM) of chromatographic samples is a greener method to both understand and evaluate a separation by scaling to the ultimate detection limit of an individual molecule. Although not as ubiquitous or versatile as conventional chromatography, SMM is a bottom-up method that uses the sensitivity of fluorescence microscopy to detect individual molecular analyte dynamics at or within the mobile/stationary phase interface.^31–33^ Anomalous events can be identified that lead to fronting, tailing, or other challenges in separations.^33–38^ In contrast to bulk elution profiles, where these behaviors can be hidden within the ensemble, the physical mechanisms that underlie a separation can be more directly probed. Recently, we have applied a 3D super-resolution fluorescence imaging method to study μm-diameter commercial stationary phases.^39^ We compared our microscopy results with ensemble HPLC of the exact same materials to show that single-molecule kinetic analysis directly predicted elution profile trends.^40^ The underlying physical mechanism of mass transfer limitations due to pore occlusion by stationary phase coatings was identified. This demonstrates that single-molecule microscopy is a tool that can help evaluate chromatographic resins prior to industrial scale-up and application, providing complementary data that could be used to enhance subsequent conventional separations. Miniaturization down to a microscopic “column” with ∼μg of stationary phase, ∼mL of solvent, and ∼nM concentrations of analyte is possible using SMM. While these numbers qualitatively sound like SMM is a greener approach, there is no standardized metric to quantify this potential performance improvement.

Here, the unified *AMGS* (*uAMGS*) is introduced to determine the greenness of the SMM of chromatography in comparison to traditional HPLC. The original *AMGS* metric is first presented with some mathematical and practical limitations for translating the approach to nonstandard separations highlighted. Accordingly, the goals in this work are 2-fold: (1) a unified *AMGS* (*uAMGS*) is presented, where all variables and mathematical expressions contributing to the precision of the greenness are explained and can be applied to any liquid separation. (2) *uAMGS* is employed to show that SMM screening improves greenness by nearly a factor of two compared with an equivalent method run on HPLC. We discuss persisting limitations of greenness metrics, including the continued dimensional inconsistencies of the original *AMGS*, resulting in part from the addition of mass and energy terms and maintained here in *uAMGS* as a heuristic measure to follow the ASC GCI Pharmaceutical Roundtable format. Finally, we present potential future directions for *uAMGS*. Through this work, we have demonstrated the applicability of the *uAMGS* to liquid-based separation techniques beyond traditional chromatography and provided clear and detailed explanations of the underlying mathematics and analysis of the uncertainty.

## Theory

### Summary and Perspective of the Analytical Method Greenness Score

The original report describing the *AMGS* ^13^ combined the safety, health, and environmental (*SHE*) assessment and cumulative energy demand (*CED*) for the solvents employed, as compiled by Henderson et al.,^41^ in addition to the energy consumption by the instrument. These three factors were scaled linearly with the combined total mass of the solvents used and inversely to the number of analytes in each trial. The original *AMGS* is repeated here, exactly as published, for reference

$$\mathrm{AMGS}=,\frac{\left(mSn+mIn\right)\times \left(3\sqrt{Sn\times Hn\times En}+CEDn+\left(Ei\times R\right)\right)}{no.analytes\;of\;interest}$$ 1

where *mSn* is the mass of solvent *n* used for preparing a standard or sample, *mIn* is the mass of *n* solvent used by the instrument during elution at a specified flow rate, *Sn*, *Hn*, and *En* are the safety, health, and environmental index scores, *CEDn* is the cumulative energy demand for each solvent, *Ei* is the instrument energy consumption, and *R* is the number of sample injections.

Equation 1 is accessible as a calculator hosted by the ACS Green Chemistry Institute Pharmaceutical Roundtable at https://acsgcipr.org/amgs/.^22^ We assume that the online calculator uses eq 1 to determine a greenness score, although all inputs are based on mL, flow rates, and run times, in contrast to units of mass for *m*S*n* and *m*I*n*. Without access to the code underlying the calculator, we are unable to determine the exact mathematical agreement of the online calculator with eq 1 but assume that densities are used. The calculator also includes acronyms and terms such as “SST” and “Soin” that, while they may be the convention within industrial use, are not defined in the original report.^13,22^ The *AMGS*calculator, therefore, may be useful for repetitive HPLC or SFC trials with commonly available instrumentation, especially in an industrial setting, but it is not more broadly applicable to methods outside traditional chromatography methods. For example, the parameters of SMM do not neatly match those in the calculator, importantly including the choice of technique itself from which the instrument energy score is calculated.

The original *AMGS* equation also has limitations and errors in its mathematical expression. First, *AMGS* primarily functions as a comparison metric between methods, as the values are not normalized. To provide a relative scale, Hicks et al. reported *AMGS* values ranging from 82.10 to 6412.27; an assessment of the literature found values reported as low as 37.99 and high as 50,513.^13^ Second, there are a few errors in the published mathematical expression: a misplaced bracket, as noted by Armstrong and colleagues,^24^ the factor of 3 in front of the *SHE* term which was meant to be a cube root to take the geometric mean of the *SHE* values, as corrected by Hashim et al. in the correct International Organization for Standardization (ISO) notation,^23,42,43^ and the potential lack of a summation to account for different solvents. Finally, the *AMGS* calculator and the use of eq 1 in the literature do not include any measure of the precision of the greenness score, which is a critical metric to understanding the statistical significance of values being compared. Based on these challenges in the *AMGS*, we present our resulting unified *AMGS*, *uAMGS*.

### Calculating the Greenness of Analytical Methods with uAMGS

The *uAMGS* represents the greenness of an analytical method based on the contributions of the instrument’s energy usage, the average *SHE* of the solvent, and energy to produce and dispose of the solvents, scaled by the number of trials and information obtained for the number of analytes. This is qualitatively represented in eq 2

2 $$\mathrm{uAMGS}=\frac{\mathrm{replicates}}{\mathrm{information}\;\mathrm{obtained}},\left[\mathrm{energy}\;\mathrm{instrument}+{\left(\bar{\mathrm{SHE}}+\mathrm{CED}\right)}_{\mathrm{solvent}s}\right]$$

Introducing quantitative variables to eq 2 in a succinct mathematical format to account for solvents involved in the separation

$$\mathrm{uAMGS}=,\frac{R_{I}}{p\times A}\left(\varepsilon_{I}+\underset{n}{\sum }\left[m_{n}\times \left(\sqrt[{3}]{S_{n}H_{n}E_{n}}+\frac{{\mathrm{CED}}_{n}}{3.6\;MJ/kWh}\right)\right]\right)$$ 3

In this equation, the total greenness score *uAMGS* is reported in units of kWh/analyte (note that this equation does not include sample preparation; see SI eq S1). The *uAMGS* is scaled by the number of replicates (*R*_I_) run on the instrument for the data set (e.g., for LC *R*_I_ = 3 to produce three replicate chromatograms to build reproducibility and statistical significance of the results of the same desired separation). The *uAMGS* is inversely proportional to the amount of information obtained, specifically the number of analytes determined, *A*, within the trial and the number of elution profiles, *p*, produced by a single replicate (e.g., for LC, *p* = 1 for one elution profile per run and for SMM *p* = 3 as three elution profiles were acquired per replicate run). The energy use of the instrument for each replicate, in kWh/replicate, is denoted by *ε*_I_. For each solvent, *n*, used, the *SHE* and *CED* terms are scaled by the solvent mass, *m*_*n*_, used in kg per replicate. The safety, health, and environmental index of each solvent is denoted by *S*_*n*_, *H*_*n*_, and *E*_*n*_, respectively, and discussed more below. Finally, *CED*_*n*_ is the cumulative energy demand metric for each solvent, *n*, based on the energy required to produce and dispose of the solvent. As *CED* possesses units of MJ/kg solvent,^44,45^ this value is divided by 3.6 MJ/kWh to match the energy units of kWh (a more detailed description of the dimensional analysis of eq 3 can be found in Table 1, eq 4, and the SI). To keep consistent with the original *AMGS* report, both the *SHE* and *CED* values were obtained from the Supplementary Table on Page 3 of Hicks et al.,^13^ which appear to be obtained from the GSK solvent selection guide reported by Henderson et al.^41^ Further updated values were reported by Alder et al.,^46^ which also group the waste into the geometric mean for a composite score. Here, *CED* remains separate from the mean *SHE*, as the production and disposal of the solvent must be done regardless of the *SHE* for a given volume of solvent.

$$\mathrm{uAGMS}\;\mathrm{units}=\frac{\mathrm{replicate}}{\mathrm{analyte}},\left(\mathrm{kWh}/\mathrm{replicate}+\underset{n}{\sum }\left[\mathrm{kg solvent}/\mathrm{replicate}\times \left(\frac{\mathrm{MJ}/\mathrm{kg solvent}}{3.6\;\mathrm{MJ}/\mathrm{kWh}}\right)\right]\right)$$ 4

**Table 1:** Unit Analysis of *uAMGS*Equation

variable	definition	unit
*R*_I_	number of replicates	replicate
*p*	number of elution profiles	unitless integer
*A*	number of analytes determined	analyte(s)
ε_I_	energy use of instrument per replicate	kWh/replicate
*m*_*n*_	mass of solvent, *n*, used for one replicate	kg solvent/replicate
*S_n_H_n_E_n_*	safety, health, and environmental indices of solvent *n*	unitless
*CED*_*n*_	cumulative energy demand of solvent, *n*	MJ/kg solvent
*uAMGS*	unified Analytical Method Greenness Score	kWh/analyte

The safety, health, and environmental indices of each solvent (*S*_*n*_, *H*_*n*_, and *E*_*n*_, respectively) are unitless values that account for several relatively scaled hazardous properties.^41,46^ For example, *S*_*n*_ combines release potential, fire/explosion risk, reactivity/decomposition, and acute toxicity, which are each relatively scaled from 0 to 1, given their different or lack of units, and then consolidated for a total *S*_*n*_ value. Each *S*, *H*, and *E* metric combines a different number of hazardous properties (e.g., *S*_*n*_ incorporates four properties while H_*n*_ incorporates two). Finally, to combine *S*, *H*, and *E* for a given solvent in the *uAMGS* equation, the cubed root of the product is used, as this geometric mean reduces the *SHE* to a single value that is less sensitive to outlying values between *S*, *H*, and *E* than an arithmetic mean (*SHE* values range from 0.01 to 4.87 for typical solvents^13^). The geometric mean of *SHE* for a given solvent is then scaled to the amount of that solvent used. Due to both the lack of units for the *S*, *H*, and *E* terms and the inconsistency in how each is determined, presenting *SHE* in terms of energy units is not currently possible. Therefore, dimensional analysis of the *SHE* term is not included in *uAMGS* (eq 4); this limitation and future work are discussed in the Limitations and Future Directions of *uAMGS* section of this manuscript.

Given the past lack of precision reported in the literature for the greenness of separations, we explicitly provide instructions for reporting the precision of *uAMGS*. The *S*_*n*_, *H*_*n*_, *E*_*n*_, and *CED*_*n*_ terms are obtained from the reference table in Hicks, which does not include precision in the measurement but are reported to one to five significant figures;^13^ we assume that these values are relatively precise and will not significantly contribute to the overall uncertainty. *R*_I_, *p*, and *A* are set integers. Therefore, the largest contribution to the total uncertainty in the reported *uAMGS* value is in ε_I_ and *m*_*n*_; the precision of these should be recorded or estimated during an experiment. For the precision of ε_I_, the energy use of the instrument should be recorded over multiple uses of the instrument (see Materials and Methods section). For *m*_*n*_, uncertainty in the volumes and/or the precision of the HPLC setup or flow system can be used to determine the uncertainty of the volumes dispensed, which are then multiplied by the density of solvents. Using these values and propagation of uncertainty of the mathematical operations^47^ in eq 3, the simplified uncertainty in the *uAMGS*, *e*_uAMGS_, is given as

5 $$e_{\mathrm{uAMGS}}=\frac{R_{I}}{p\times A}\sqrt{{\left(e_{\varepsilon_{I}}\right)}^{2}+\sum_{n}{\left[e_{m_{n}}\times \left(\sqrt[{3}]{S_{n}H_{n}E_{n}}+\frac{{CED}_{n}}{3.6\;MJ/kWh}\right)\right]}^{2}}$$

where *e*_ε_I__ and *e*_*m*_*n*__ are the uncertainty in the energy use of the instrument and the solvent (*n*) masses, respectively, and other variables are as defined above. The final *uAMGS* value should then be reported as

6 $$\mathrm{uAMGS}\pm e_{\mathrm{uAMGS}}$$

with units of kWh/analyte and a correct number of significant figures^48^ in *uAMGS* given by the traditional rounding rules of the uncertainty in *e*_uAMGS_.

## Materials and Methods

### HPLC Experimental Conditions and Variable Values

Values of solvent use are based on data collected as previously described in Monge Neria et al.,^40^ and energy consumption values are new to this work. All HPLC experiments were performed on a Shimadzu liquid chromatography instrument (LC-20AD, Prominence) with a REFLECT c-Cellulose-B (5 μm, 5 cm × 4.6 cm from Regis Technologies) column and photodiode array detection (SPD-M20A, Prominence).

A 100 μM rhodamine 6G sample in aqueous HEPES buffer was prepared by dilution of a 25.00 mL aqueous stock to a 2.00 mL working standard, leading to a total sample solvent volume of 27.00 ± 0.17 mL water (0.02700 ± 0.00017 kg). The 100 μM rhodamine 6G standard in aqueous HEPES buffer was injected (3.0 μL) on the column with an isocratic mobile phase of 70% ethanol (200 proof, ACS grade from Pharmaco) and 30% aqueous HEPES (20 mM, pH 7.33, 2-[4-(2-hydroxyethyl)-1-piperazinyl]-ethanesulfonic acid, high purity, VWR), a flow rate of 0.5 mL/min, a run time of 10.0 min, and a column temperature of 30 °C. Detection was accomplished using a photodiode array over the range 200–600 nm at 5 Hz. Using flow rate and run time, these conditions resulted in a calculated 3.5 ± 0.4 mL (0.0028 ± 0.0003 kg) of ethanol and 1.5 ± 0.2 mL (0.0015 ± 0.0002 kg) of water used per elution by the HPLC. To take into account HPLC cycle time, HPLC solvent consumption was also directly measured by collecting the eluate over the course of one experiment (consisting of four replicate injections) and determining the volume of each solvent from the set percent composition, resultant mass of collected eluate, and solvent densities, per injection. This process was performed in triplicate and resulted in 4.04 ± 0.07 mL (0.00319 ± 0.00006 kg) of ethanol and 3.18 ± 0.06 mL (0.00318 ± 0.00006 kg) of water used per elution.

To determine energy consumption of the HPLC, all LC modules, diode array, and computer were routed through a power conditioner (Powervar Ametek 8.3), which was plugged into a power consumption monitor (Fayleeko, PMP01) that recorded the kWh and time over the course of an experiment (consisting of start-up, 10 min purging, 4 replicate injections, and power down). The power monitor was plugged into PowerVar Security Plus II UPS, which in turn was connected directly into the house electrical. To determine the average energy consumption per run, this total was divided by the number of runs performed while undergoing monitoring. This experiment was performed in triplicate to determine the average energy consumption and standard deviation of 0.080 ± 0.010 kWh/replicate.

### Single-Molecule Microscopy Experimental Conditions and Variable Values

Quantitative values of solvent and energy use are based on single-molecule microscopy data collected as previously described in Monge Neria et al.^39,40^ Briefly, commercial stationary phase particles were immobilized on cleaned silica glass coverslips for microscopy. All uses of water are Type 1 ultrapure water purified on an Elga Chorus 2+ system. Different suspensions of 0.1 wt % solid stationary phases were used to deposit cellulose tris (3,5-dimethylphenylcarbamate)-functionalized fully porous 5.0 μm silica stationary phase particles (Regis). An 8 μL volume of the stationary phase solutions was then dropcast and physisorbed to the slides by drying in ambient air. The samples were then covered by silicon flow cells (Hybriwell, 13 mm diameter, 0.15 mm depth, Grace Biolabs) and rinsed multiple times with water, then left to rehydrate for a minimum of 2 h before imaging.

Single-molecule microscopy was performed in a highly inclined and laminated optical sheet (HILO) fluorescence imaging of nanomolar concentrations of fluorescent rhodamine 6G dye (99%, Fisher) under flow. The instrumentation includes a home-built microscope that consists of an Olympus IX-73 inverted microscope, a CNI diode laser (532 nm, MGL-III-100 mW) for excitation, a *z* (vertical) single axis translation nanopositioner (Physik Instrumente, PIFOC, P-725) paired with the 100× magnification oil immersion objective (Olympus, 100×, NA 1.49, UAPON100XOTIRF), and an EMCCD camera (iXon Life 897, Andor) to collect the emission signal. Imaging was done with 1 nM rhodamine 6G solutions in 20 mM HEPES buffer (Biotang Inc., pH 7.33), passed through the flow cells at a rate of 5.0 ± 0.1 μL/min using a syringe pump (NE-1000, New Era Pump Systems Inc.). Typical experimental time for multiple trials within and between different samples is ∼1 to 4 h, where millions of single-molecule adsorption events are collected over tens or hundreds of stationary phase particles. Specifically for our data here, each individual sample is imaged in three different areas with multiple stationary phase particles to produce three elution profiles (*m*) within 30 min. Three replicate (*R*_I_) samples are measured to produce three elution profiles per sample for nine total elution profiles within 90 min (time is inclusive of experimental setup, determining the best field of view, equilibration, and active data collection). The total use of solvent is then 0.45 ± 0.01 mL of water (4.5 ± 0.1 × 10^–4^ kg).

To determine the energy use, ε_I_ of the SMM instrumentation, a power consumption monitor (Fayleeko, PMP01) recorded the kWh consumption, time, power, and maximum and minimum power over the course of an experiment. For microscopy, all electronic components, including laser power supply, camera, computer, and syringe pump, were plugged into a single surge protector that was then connected into the power meter supplied by the 120 V AC house electrical. At least three trials were recorded to obtain the average and standard deviation in energy of 0.172 ± 0.022 kWh/replicate as used in eqs 1 and 3.

## Results and Discussion

### Greenness Comparison Between HPLC and SMM Methods Exemplifies Uses of *uAMGS*

The greenness of the same analyte and the same stationary phase materials analyzed both with HPLC and SMM is determined and compared. In our previous research,^40^ we studied a liquid chromatography sample of rhodamine 6G fluorophore as the analyte on a cellulose-B stationary phase in an aqueous-based mobile phase. Rhodamine 6G can be detected by both absorbance detection and, due to its high fluorescence quantum yield, via emission at the single-molecule level by microscopy. Importantly, we previously showed that single-molecule mass transfer analysis of the desorption can directly predict HPLC elution profile behaviors (Figure S1).^40^ We were the first to show this agreement by measuring the same exact chromatography material on the single-molecule scale on our microscope and within an HPLC column and observed similar trends between the two systems despite their vastly different scales (from μm to cm). The agreement between a column-scale, bulk HPLC separation and single-molecule data therefore ensures that an equivalent comparison is made in our *uAMGS* calculation, while highlighting fundamental differences between *uAMGS* and *AMGS*.

A conventional *AMGS* calculation shows that both HPLC and SMM chromatography of rhodamine 6G are relatively greener methods when compared with the range of typical *AMGS* calculations. Using eq 1, as published by Hicks et al.,^13^ while assuming masses in g and summation across solvents inclusive of *mSn* to *CEDn* values, resulted in values of *AMGS*_*HPLC*_ = 76.27 and *AMGS_SMM_* = 1.23 (Figure 1A,B; x marks), reported to two decimal places as was done in the original report. Regardless, all *AMGS* values are relatively low compared to ranges in the original report of 82.10–6412.27 (Figure 1A,B; dashed lines). Given the large disparity between the two methods, a log scale in Figure 1B better represents the differences in the greenness. The SMM value is well below the range of the original report, highlighting how miniaturization improves the greenness. Interestingly, the *AMGS_HPLC_* from eq 1 (76.27) differs from the value calculated from the *AMGS* online calculator^22^ of 62.21 (Figure 1A,B; circle), likely due to the mathematical errors described above or aspects of the calculator not present in eq 1. As the online calculator only lists various conventional chromatography methods and has limited inputs,^22^ *AMGS_SMM_* cannot be determined using this tool. Overall, the inconsistencies in the values, unknown uncertainties, and inability to use the online calculator to determine an SMM value led to our subsequent calculation via *uAMGS*.

The unified *AMGS* quantifies that the SMM of chromatography is greener than conventional HPLC due to reduced amounts of solvents and energy consumption of instrumentation per information obtained. Using eqs 3 and 5, *uAMGS* scores of 0.301 ± 0.026 kWh/analyte and 0.172 ± 0.022 kWh/analyte for HPLC and SMM, respectively, were determined. These *uAMGS* values are significantly different (*p* = 0.0028, two-tailed *t*-test), which can be determined due to the incorporation of uncertainty into *uAMGS*. These values are visualized in Figure 1C (linear axes) and 1D (log axes). In this study, we elected to take *R*_I_ = 3 and *p* = 3 for the SMM data (i.e., three replicates and three elution profiles per replicate for a total of nine elution profiles); however, significantly more data are collected in a single SMM run. The many hundreds of stationary particles in a single field of view, if analyzed, would substantially increase *p* and decrease *uAMGS* (e.g., if *p* = 250, then *uAMGS* = 0.0021 kWh/analyte). Finally, to highlight the differences in energy consumption due to instrumentation (ε_I_ term) and solvent contributions (*SHE* and *CED* terms), the percentage of each was determined via SI eqs S10 and S11 and reported in Figure 1E.

Energy consumption due to solvent use is the main contributor to the difference in *uAMGS* scores between SMM and HPLC. As indicated in Figure 1E, HPLC solvent use is the major factor for our analytical scale chromatography trial, despite only requiring 5.0 mL (or 7.2 mL including cycle time solvent use, discussed below) of an ethanol/water solvent mixture per replicate. In contrast, the reduced sample volume requirement of SMM due to the 20 mm^3^ sample cell necessitating only 0.45 mL of a water solvent to collect many replicates, and millions of single-molecule events significantly reduced the solvent’s contribution to the greenness score to negligible values. The improvement in greenness by reducing solvent consumption and waste disposal in SMM is in line with broader approaches in method miniaturization (e.g., microfluidics^49–51^ ) and, more specifically, advancements in liquid chromatography to decrease mobile phase flow rates.^52^ Prior research has demonstrated LC-based separations on reduced internal diameter columns (e.g., 150 μm) and μL/min flow rates with custom instrumentation, which achieve a reduction in solvent use by 1000-fold.^53^ Our SMM work here similarly reduces volumetric flow rate by 2 orders of magnitude from 0.5 to 0.005 mL/min with a concomitant reduction in solvent volume.

**Figure 1:**
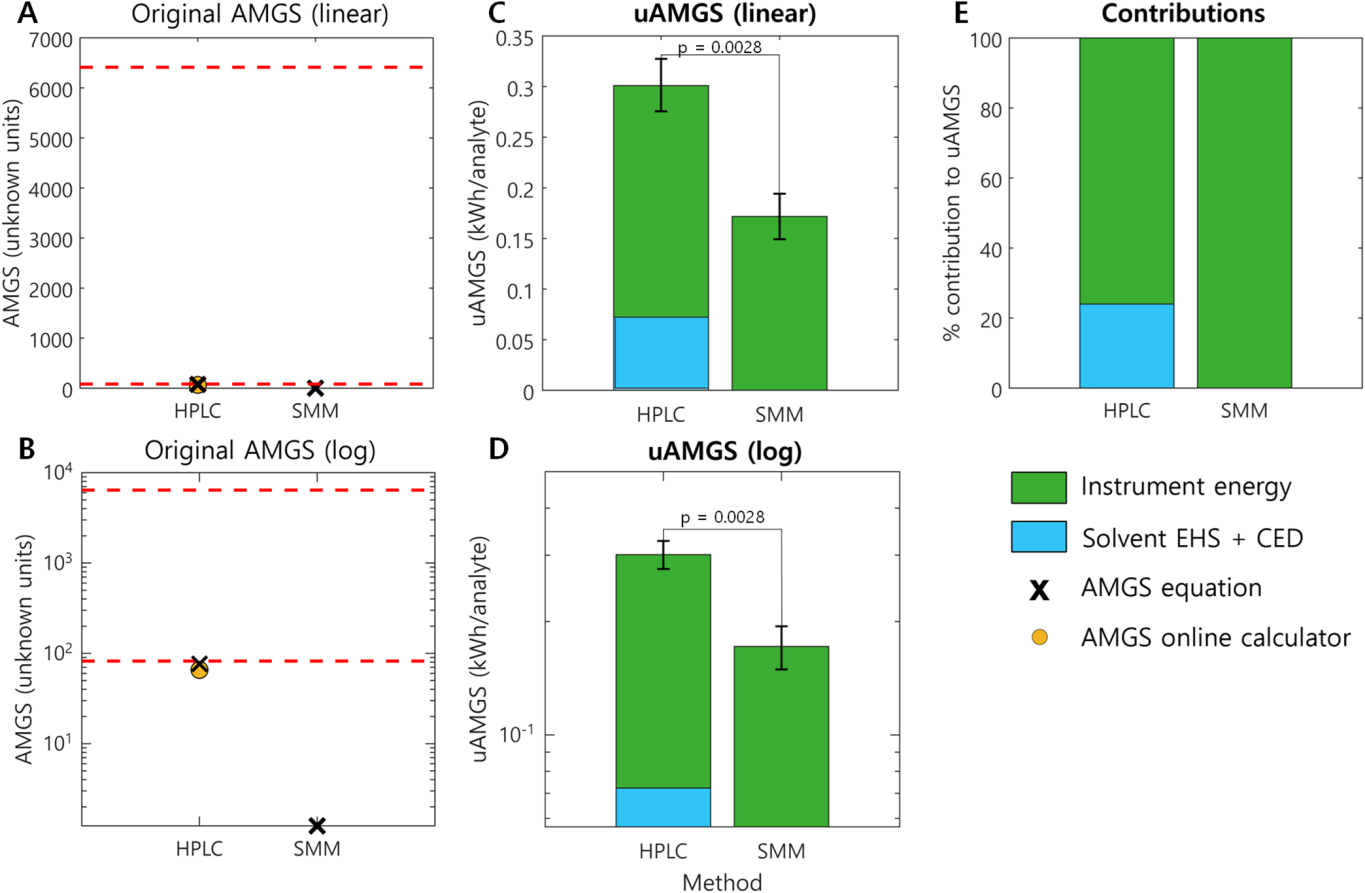
*uAMGS* provides more information about the improved relative greenness between conventional HPLC and single-molecule microscopy (SMM) of liquid chromatography materials and analyte elution. Data obtained from rhodamine 6G analyte on cellulose-B stationary phase. (A, B) *AMGS* values calculated using eq 1 (black X) from Hicks et al. for HPLC and SMM, and the *AMGS* online calculator for HPLC (orange circle), compared relative to the minimum and maximum reported *AMGS* values (red dashed lines). No measure of precision is provided, so no error bars are present in the single values reported. Values are reported on (A) linear and (B) log-based 10 axes without units, consistent with eq 1 from the original report. (C, D) Unified *AMGS*(*uAMGS*) of HPLC and SMM reported on (C) linear and (D) log-based 10 axes with units of kWh/analyte as derived in eqs S4–S9 and error bars representing the absolute uncertainty as described in eqs 5 and 6. (E) Percent contributions of the energy the instrument uses (ε_I_ term, green) and energy consumption due to the solvent (*SHE* and *CED* terms, blue) to the *uAMGS*.

Inclusion of HPLC cycle time solvent use impacts the *uAMGS* value, although not significantly in these experiments. The effect of HPLC cycle time, or the time between the end of one chromatographic run and start of the next during which the instrument is equilibrating and consuming solvent, on *AMGS* has previously been discussed by Handlovic et al..^24^ Here, we compare *uAMGS* values for HPLC determined using both the calculated solvent consumption (using flow rate and run time) and measured solvent consumption (which includes cycle time) as described in the Materials and Methods. While the *uAMGS* value that incorporates cycle time solvent consumption is higher at 0.311 ± 0.025 kWh/analyte compared to 0.301 ± 0.026 kWh/analyte (from calculation, as reported above), these values are not significantly different (*p* = 0.7, two-tailed *t*-test). As these HPLC experiments were via isocratic elution, there was no need for a prolonged equilibration period between runs. The cycle time was therefore minimized, and the difference between the measured and calculated solvent consumption was relatively low, resulting in an insignificant difference in *uAMGS* values. Methods that have a significant cycle time, especially relative to the overall run time, should consider the additional solvent consumption when determining *uAMGS*, either by adding the cycle time to the run time in the solvent calculation^24^ or, as was done here, by measuring the HPLC eluate. HPLC required slightly less energy use for instrumentation (0.080 ± 0.008 kWh/replicate) than SMM (0.172 ± 0.022 kWh/replicate). This absolute difference in energy consumption is likely due to the longer time frame that is required for an SMM replicate in this work (1.5 h for SMM vs 10 min for HPLC), although as noted above the amount of data obtained for a single replicate using SMM (three elution profiles per replicate) vastly outpaces HPLC (one elution profile). Additionally, in the future, the time frame for SMM measurements could be reduced as the active measurement time is on the order of a few minutes; in this study, very careful, ideal measurements were taken that required more preparation time than typical for more routine data acquisition. Finally, we note that the original *AMGS* reported HPLC instrumentation using substantially more energy consumption (0.644 kWh^13^) came with large assumptions (40% of listed energy requirements); this large difference between the two HPLC instruments clearly demonstrates the need for energy measurements to be made on the specific instrument and not approximated when employing greenness calculations. Overall, SMM is a greener method than HPLC as determined using *uAMGS*.

### *uAMGS* is Accessible to Research Beyond Traditional Chromatography and as a Green Chemistry Instruction Tool

The initial *AMGS* report was primarily focused on pharmaceutical industry applications involving analysis using conventional chromatography. Others have found that “most workers find the [*AMGS*] program difficult to handle and maintain because it demands a large amount of information entry.”^54^ In contrast, *uAMGS* is a tool that can be used by researchers doing liquid separations outside of the pharmaceutical industry and conventional chromatography. We have demonstrated its utility in SMM; we believe that there are other methods, including paper-based separations,^55,56^ preparative separations,^57,58^ membranes,^59,60^ MOF/COFs,^61^ and microfluidics,^25,49–51^ where *uAMGS* as presented could be directly applied. We have outlined the variables and the mathematics behind *uAMGS* so that it can be more widely employed by those outside of chromatography users in industry. Further, *uAMGS* can be applied to new technologies which may require additional considerations beyond those already incorporated. Additional additive or multiplicative terms could be included to accommodate other experimental contributions to greenness that are specific to those methods.

The *uAMGS* can also be used as a teaching tool to promote discussion of green chemistry principles. ACS lists as a critical requirement in its program accreditation that the “curriculum must provide students with a working knowledge of the Twelve Principles of Green Chemistry.”^62^ The presentation of the relative weight and mathematical reasoning behind each of the components in eqs 2–5 can be used as an instructional tool as early as the undergraduate level, enabling the next generation of chemists to think more deeply about green principles. Equations 2 and 3 clearly showcase the contributions of safety, health, and environmental risks of the solvent (*SHE*), cumulative energy demand of the production and disposal of the solvents (*CED*), and energy use of the instrument, which can be used in green chemistry discussions. Specifically, aspects of employing less hazardous chemicals, safer solvents, and instrumentation design for improved energy efficiency, all of which are captured by the *uAMGS*. Reasoning behind mathematical operations and a discussion of units (SI) were included for students who may be using this tool. Finally, although greenness is often discussed using subjective and qualitative descriptors, scientists should strive for comprehensive quantitation, including a metric’s uncertainty. Equations 5 and 6 detail how the uncertainty in *uAMGS* can be determined using replicate measurements and standard propagation of uncertainty,^47^ allowing statistical significance to be determined when comparing two methods.

Here, *uAMGS* has been applied for both HPLC and SMM methods; however, we believe that this calculation is applicable to other separations, even those using paper- or TLC-based methods. As the mathematical approach to *uAMGS* has been clearly laid out, instructors and students can feel confident in modifying *uAMGS* to investigate greenness beyond separations to techniques such as flow injection analysis or even atomic absorbance spectroscopy. We encourage comparisons between methods on the same instrument and/or between different instruments as part of undergraduate laboratory courses.

### Limitations and Future Directions of *uAMGS*

Like many other greenness calculators, *uAMGS* is best used for comparisons between methods and techniques and not as an absolute energy equivalent to an International System of Units (SI).^54^ While the present work makes advances toward an absolute energy consumption metric by clarifying terms and units, *uAMGS* functions best as a comparative tool between multiple methods, techniques, or operating conditions due to how select inputs are determined, like the original *AMGS*. Other methods for determining greenness, including the Analytical GREEnness metric and software (AGREE)^17^ and related calculations,^18,19^ avoid this challenge through scaling of the final result from 0 to 1; however, normalization lacks a physical meaning. Here, units of kWh/analyte were maintained to strive for a measure of energy use, but future considerations of the *SHE* of the solvents, as discussed below, would be needed to achieve a complete physical representation of the energy consumed for all aspects of the method.

The *uAMGS* method can be applied not only to compare multiple methods’ relative greenness but also to determine the relative greenness of different optimization approaches.^63–66^ Here, two already optimized methods’ metrics of greenness were compared; only the direct experimental conditions used to acquire publishable data were included in the *uAMGS* calculations. In the future, or for any method that is in the development stage, the contributions to *uAMGS* for all optimization steps could be incorporated into a final reported *uAMG*S value. For example, an evaluation of the design of experiment, chemometrics, and multiple response optimization choices could be achieved. Finally, the *uAMGS* metric takes into account the number of instrumental and sample replicates (eq 3 and SI eq S1). By incorporating these replicates, *uAMGS* can make greenness comparisons between two methods or techniques that may fundamentally require a different number of replicates to achieve statistically significant data or results.

A limitation of *uAMGS* is that it does not account for the consumption of consumables (e.g., vials, pipette tips, syringes, etc.), computer energy used in analysis, or specific chemicals (e.g., derivatization reagents, buffers, etc.). It also does not account for differences in equipment manufacture, installation, or maintenance. Some of these items are comparable across methods, difficult to make greener, and/or challenging to assess in the lab environment. There is a plethora of ways to calculate greenness,^16–21,67^ many of which make the same compromises highlighted here.

Future work can provide further information and transparency on the *SHE* of the solvents. Each *S*, *H*, and *E* index are themselves combinations of several relatively scaled (from 0 to 1) unitless hazardous properties. The value of *SHE* for each solvent used here is the exact same value provided in the SI of Hicks et al.,^13^ who adapted the values from the solvent selection guide,^41^ although there is a lack of clarity on how these values were calculated in Hicks et al. (some appear as a sum while others do not). Assuming a sum is used from the different categories (e.g., two categories of irritation and chronic toxicity for *H* or four categories of release potential, fire/explosion, reactivity/decomposition, and acute toxicity for *S*), the final values could have a higher weighting of values with a greater maximum. Therefore, future work could develop a more consistent procedure for the calculation of the final values of *S*, *H*, and *E*, better allowing the reported *SHE* value to more clearly represent each factor in the final *uAMGS* metric.

One major limitation of both the *AMGS* and now *uAMGS* is how the *SHE* term is incorporated into the metric regarding units. Here, *uAMGS* maintains the same overall contribution of terms as the original *AMGS* for consistency, specifically summing the effects of the instrument energy, *SHE* of the solvents, and *CED* of the solvents (eqs 1–3). The reasoning is that each of these factors is an important contributor to a method’s overall greenness. *SHE* and *CED* are summed and scaled by the mass of solvent to have a term that specifies the contribution of the solvents to the greenness. This rationale unfortunately creates a complication in dimensional analysis: currently, *SHE* is unitless and is inherently not an energy metric; therefore, it cannot be reported in units of energy,^13,41^ unlike the other two terms, *ε*_I_ and *CED*_*n*_. As a result, *SHE* was not included in the dimensional analysis of *uAMGS* (eq 4), although it was included in the overall *uAMGS* metric. Potential future work to solve this limitation could be to improve the calculation of the *SHE* term, as discussed above, or isolate the *SHE* term as a completely independent term or metric to consider energy and safety/health/environmental cost separately. We encourage a continued discussion by the field and the ACS GCI Pharmaceutical Roundtable, especially focused on a quantitatively robust, absolute metric with recognized units related to *SHE*.

## Conclusions

A unified analytical method greenness score was developed that has broad applicability beyond traditional chromatographic separations and the pharmaceutical industry. The underlying mathematics, precision, and unit analysis for this calculation were clearly described. *uAMGS* was determined for two equivalent methods, SMM and HPLC, demonstrating that SMM is a significantly greener method. Overall, this work breaks down some of the different factors that should be incorporated into greenness, the relative importance of each factor, and their limitations. Finally, we have shown that *uAMGS* captures the benefits of miniaturization, orthogonality, and multiplicity in cutting-edge measurement science.

## Supplementary Material



## Notes

*uAMGS* equations that incorporate sample preparation, a discussion of dimensional analysis, the determination of the percent energy contribution of the solvent vs. instrument energy consumption, and example data from the separations compared in this manuscript (PDF)
