# Supplementary material for: A Unified Analytical Method Greenness Score (uAMGS) Quantifies How Microscopic Imaging Is Greener Than Conventional Liquid Chromatography
Source: ACS Sustain Chem Eng. 2026 Jun 6;14(24):11058–67. doi: 10.1021/acssuschemeng.6c02093 (PMC13292395; doi:10.1021/acssuschemeng.6c02093)
Supplement: Supplementary file 1 [file sc6c02093_si_001.pdf]

SUPPORTING INFORMATION

A unified analytical method greenness score (*uAMGS*) quantifies how microscopic imaging is greener than conventional liquid chromatography

Kotomi Inaba,[1] Ricardo Monge Neria,[2] Rachel A. Saylor[1,†,*], and Lydia Kisley,[2, 3†,*]

[1]Department of Chemistry and Biochemistry, Oberlin College, Oberlin, OH, USA 44074

[2]Department of Physics and [3]Department of Chemistry, Case Western Reserve University, Cleveland, Ohio, USA 44106

* rsaylor@oberlin.edu, lydia.kisley@case.edu

SI Table of Contents

uAMGS calculation when a sample is prepared separately from the analysis page S2
Dimensional analysis of *uAMGS* page S2
Determination of the percent energy contribution of the instrument and solvents page S4
Figure S1: SMM and HPLC experimental data page S4
Original *AMGS* calculator inputs and results for HPLC page S5
SI References page S7

uAMGS calculation when a sample is prepared separately from the analysis

The *uAMGS* equations in the main text assume that the sample is created separately from any analysis, as is the case with SMM. However, many experiments require a separate sample preparation where the sample is then injected into the instrument. In these cases, including with HPLC, the energy impact of the solvent from the sample preparation can also be determined, separately from that of the instrumental analysis. This can be seen in equation S1, with the first term focusing on sample preparation and the second the instrumental analysis as discussed in the main text:

$$uAMGS = \frac{R_S}{A}\left(\varepsilon_S + \sum_n \left[m_{n,S} \times \left(\sqrt[3]{S_n H_n E_n} + \frac{CED_n}{3.6\ MJ/kWh}\right)\right]\right) + \frac{R_I}{p \times A}\left(\varepsilon_I + \sum_n \left[m_{n,I} \times \left(\sqrt[3]{S_n H_n E_n} + \frac{CED_n}{3.6\ MJ/kWh}\right)\right]\right) \quad \text{(S1)}$$

In this equation, R_S are the sample replicates, *A* is the number of analytes analyzed, $m_{n,S}$ is the mass (in kg) of the *n* solvent(s) used in the sample preparation. The variable ε_S denotes the energy consumption, in KWh, of any equipment used to prepare the sample (e.g. heating/stir plates, microwave oven, *etc*.). All other variables are the same as those in Equation 3 of the main text. Briefly, S_n, H_n, and E_n are the safety, health, and environmental index for each n solvent, respectively, and CED_n is the cumulative energy demand of n solvent; all of these values are found in the SI of Hicks et al..[1] The energy consumption of the instrument is ε_I and the mass of the *n* solvent(s) used by the instrument is $m_{n,I}$ and should be recorded in kg. Finally, the number of replicates run on the instrument and the number of elution profiles produced by a single replicate are denoted by R_I and *p*, respectively. Note that the terms R_I, *p*, and ε_I only appear in the second instrument analysis term, as these metrics are not relevant to sample preparation.

Dimensional analysis of *uAMGS*

As presented in the main text and reproduced here, *uAMGS* can be determined as follows:

$$uAMGS = \frac{Replicates}{Information\ obtained}\left[Energy\ instrument + \left(\overline{SHE} + CED\right)_{solvents}\right] \quad \text{(S2)}$$

$$uAMGS = \frac{R_I}{p \times A}\left(\varepsilon_I + \sum_n \left[m_n \times \left(\sqrt[3]{S_n H_n E_n} + \frac{CED_n}{3.6\ MJ/kWh}\right)\right]\right) \quad \text{(S3)}$$

The variables in Equation S3 are defined both in the main text and above. We can replace these variables with the units that they represent (Equation S4) and do some preliminary unit analysis (Equation S5):

$$uAGMS\ units = \frac{\text{replicate}}{\text{analyte}}\left(\text{kWh/replicate} + \sum_{\text{n}}\left[\text{kg solvent/replicate} \times \left(\frac{\text{MJ/kg solvent}}{\text{3.6 MJ/kWh}}\right)\right]\right) \quad \text{(S4)}$$

$$uAGMS\ units = \frac{\text{replicate}}{\text{analyte}}\left(\text{kWh/replicate} + \sum_{\text{n}}\left[\text{kg solvent/replicate} \times \left(\frac{\text{kWh}}{\text{kg solvent}}\right)\right]\right) \quad \text{(S5)}$$

Equation 3 contains the conversion factor of 3.6 MJ/kWh. This factor was added to convert the units from the *CED* term (originally MJ/kg solvent) into the more conventional energy measurement of kWh/kg solvent. This change keeps the *CED* term units consistent with those of the ε_l term.

As discussed in the main text, each component of the *SHE* term is itself a combination of terms; for example, the safety index *S* accounts for a combination of release potential, fire/explosion risk, reactivity/decomposition, and acute toxicity while the health index *H* accounts for the irritation and chronic toxicity of the specified solvent. To the best of our knowledge, each individual term (e.g., acute toxicity) is denoted on a relative scale of 0 to 1 and therefore does not possess units.[1–3] In this work, the exact same values as provided in the SI of Hicks *et al.*[1] were used for consistency; however, we the lack of clarity on how these values were calculated (some appear as a sum while others do not). While it is beyond the scope of the present work, a more absolute measurement and/or more transparency and consistency in their determination for each of these terms could be considered. Due to these constraints the *SHE* units have been omitted from further consideration at the present time. With more unit analysis, Equation S5 can be simplified further:

$$uAGMS\ units = \frac{replicate}{analyte}\left[kWh/replicate + \sum_{n}\left[\frac{kWh}{replicate}\right]\right] \quad \text{(S6)}$$

$$uAGMS\ units = \frac{\text{replicate}}{\text{analyte}}\left(\text{kWh/replicate} + \sum_{\text{n}}\left[\text{kg solvent/replicate} \times \frac{\text{kWh}}{\text{kg solvent}}\right]\right) \quad \text{(S7)}$$

$$uAGMS\ units = \frac{\text{replicate}}{\text{analyte}}\left(\text{kWh/replicate} + \sum_{\text{n}}\left[\frac{\text{kWh}}{\text{replicate}}\right]\right) \quad \text{(S8)}$$

$$uAGMS\ units = \frac{kWh}{analyte} \quad \text{(S9)}$$

These final unit cancelations leave the *uAMGS* units of kWh/analyte. In other words, *uAMGS* denotes the amount of energy consumed to obtain information on a per analyte basis.

Determination of the percent energy contribution of the instrument and solvents

The percent energy contribution of the instrument (Equation S10) vs. the solvents (Equation S11) were determined via the following equations. Variables in these equations are these same as Equation 3 and defined in the main text.

$$\%_{Energy\ Instument} = \frac{\varepsilon_I}{\varepsilon_I+\sum_n\left[m_n\times\left(\sqrt[3]{S_nH_nE_n}+\frac{CEDn}{3.6\ MJ/kWh}\right)\right]} \times 100\% \quad \text{(S10)}$$

$$\%_{Energy\ Solvent} = \frac{\sum_n\left[m_n\times\left(\sqrt[3]{S_nH_nE_n}+\frac{CEDn}{3.6\ MJ/kWh}\right)\right]}{\varepsilon_I+\sum_n\left[m_n\times\left(\sqrt[3]{S_nH_nE_n}+\frac{CEDn}{3.6\ MJ/kWh}\right)\right]} \times 100\% \quad \text{(S11)}$$

SMM and HPLC experimental data

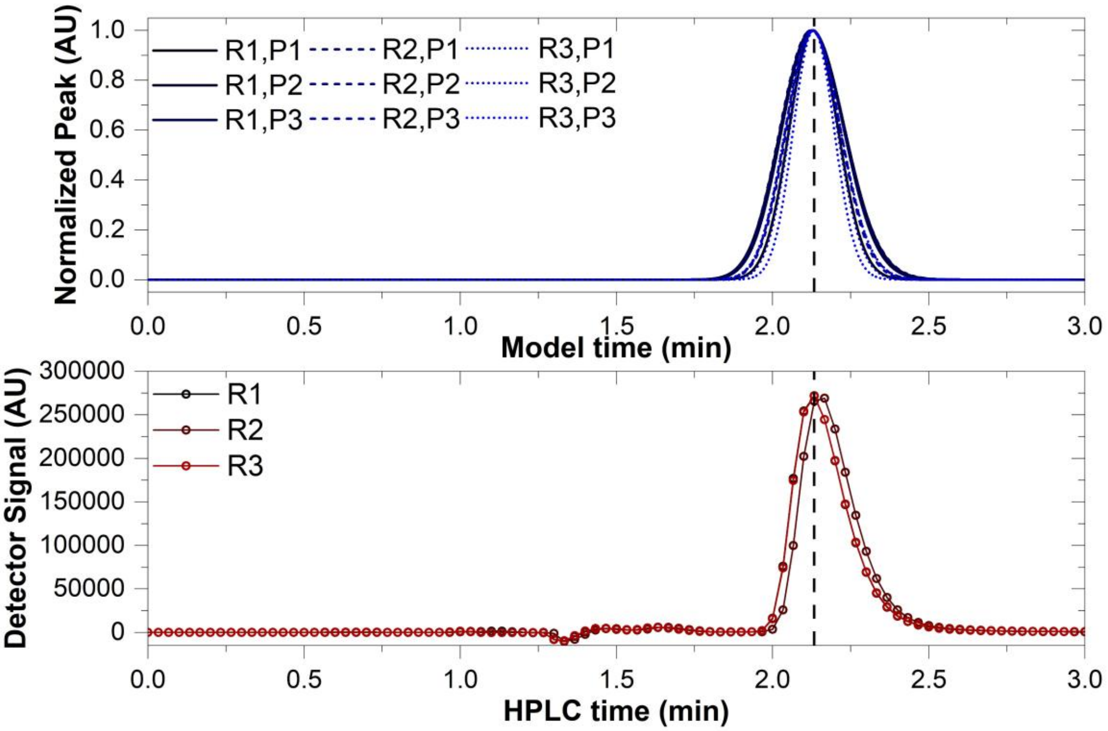

Figure S1. Single molecule microscopy data shows equivalent elution profile to conventional liquid chromatography using rhodamine 6G and a cellulose B stationary phase. Modeled elution profile from single molecule microscopy observations (blue) from three replicates each observing three chromatography particles (9 total elution profiles) and HPLC elution profile (red) from three replicate measurements. Additional experimental detail in the methods section; raw data from Monge Neria et al. (2025).[4]

Original *AMGS* calculator inputs and results

The AMGS calculator, hosted by the ACS Green Chemistry Institute Pharmaceutical Roundtable (https://www.acsgcipr.org/amgs/)[5], was used to determine the $AMGS_{HPLC}$ value of 62.21. Experimental conditions are listed in the methods section of the manuscript. The relevant inputs and results are below; unused inputs were omitted from this image.

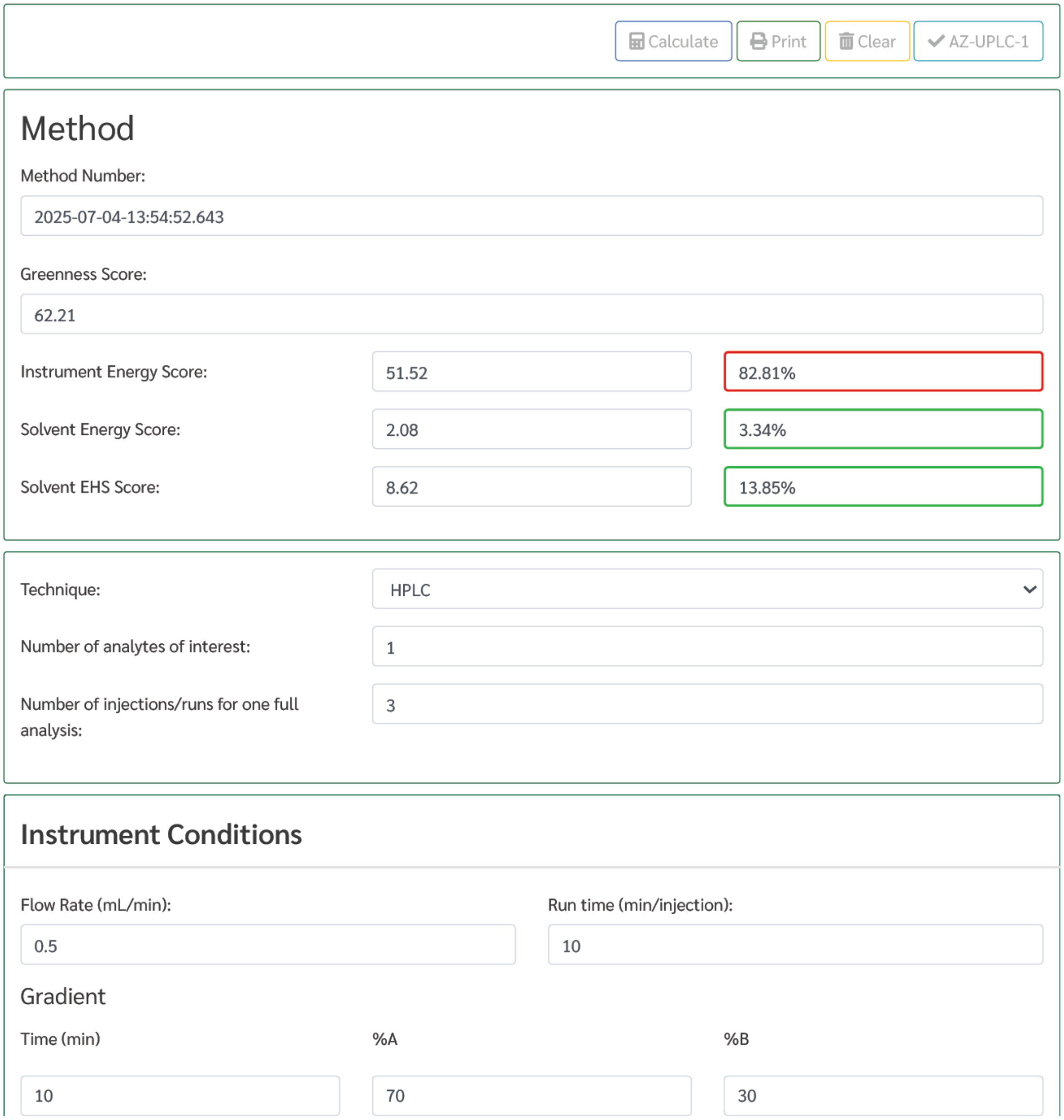

Calculate | Print | Clear | AZ-UPLC-1

Method

Method Number: 2025-07-04-13:54:52.643

Greenness Score: 62.21

Instrument Energy Score:	51.52	82.81%
Solvent Energy Score:	2.08	3.34%
Solvent EHS Score:	8.62	13.85%

Technique:	HPLC
Number of analytes of interest:	1
Number of injections/runs for one full analysis:	3

Instrument Conditions

Flow Rate (mL/min): 0.5

Run time (min/injection): 10

Gradient

Time (min)	%A	%B
10	70	30

Mobile Phases

Mobile Phase A

SOLVENT 1	SOLVENT 2	SOLVENT 3
Ethanol	-	-
Percent	Percent	Percent
100	Percent	Percent

Mobile Phase B

SOLVENT 1	SOLVENT 2	SOLVENT 3
Water	-	-
Percent	Percent	Percent
100	Percent	Percent

Sample

Sample Diluent

Sample prep volume (mL): 27

Number of sample preps: 1

SOLVENT 1	SOLVENT 2	SOLVENT 3
Water	-	-
Percent	Percent	Percent
100	Percent	Percent

SI References

(1) Hicks, M. B.; Farrell, W.; Aurigemma, C.; Lehmann, L.; Weisel, L.; Nadeau, K.; Lee, H.; Moraff, C.; Wong, M.; Huang, Y.; Ferguson, P. Making the Move towards Modernized Greener Separations: Introduction of the Analytical Method Greenness Score (AMGS) Calculator. *Green Chemistry* **2019**, *21* (7), 1816–1826. https://doi.org/10.1039/c8gc03875a.

(2) Henderson, R. K.; Jiménez-González, C.; Constable, D. J. C.; Alston, S. R.; Inglis, G. G. A.; Fisher, G.; Sherwood, J.; Binks, S. P.; Curzons, A. D. Expanding GSK's Solvent Selection Guide – Embedding Sustainability into Solvent Selection Starting at Medicinal Chemistry. *Green Chemistry* **2011**, *13* (4), 854–862. https://doi.org/10.1039/c0gc00918k.

(3) Alder, C. M.; Hayler, J. D.; Henderson, R. K.; Redman, A. M.; Shukla, L.; Shuster, L. E.; Sneddon, H. F. Updating and Further Expanding GSK's Solvent Sustainability Guide. *Green Chemistry* **2016**, *18* (13), 3879–3890. https://doi.org/10.1039/c6gc00611f.

(4) Monge Neria, R.; Zeeshan, M.; Kapoor, A.; Kim, T. K. J.; Hoven, N.; Pigott, J. S.; Gurkan, B.; Duval, C. E.; Saylor, R. A.; Kisley, L. Super-Resolution Imaging Reveals Resistance to Mass Transfer in Functionalized Stationary Phases. *Sci Adv* **2025**, *11* (7), 790–800. https://doi.org/10.1126/sciadv.ads0790.

(5) ACS Green Chemistry Institute Pharmaceutical Roundtable. *AMGS Calculator*. https://acsgcipr.org/amgs/ (accessed 2025-08-07).
